# Reduction of multiplicity of infections but no change in *msp2* genetic diversity in *Plasmodium falciparum* isolates from Congolese children after introduction of artemisinin-combination therapy

**DOI:** 10.1186/1475-2875-11-410

**Published:** 2012-12-07

**Authors:** Rod Ibara-Okabande, Felix Koukouikila-Koussounda, Mathieu Ndounga, Jeannhey Vouvoungui, Vladimir Malonga, Prisca Nadine Casimiro, Jean Rosaire Ibara, Anissa Sidibe, Francine Ntoumi

**Affiliations:** 1 Fondation Congolaise pour la Recherche Médicale, Brazzaville, Republic of Congo; 2 Faculty of Health Sciences, University Marien Ngouabi, Brazzaville, Republic of Congo; 3 Centre de Recherche sur les Ressources Végétales, Brazzaville, Republic of Congo; 4 Centre de Recherche sur le Paludisme, Hôpital de Base de Makélékélé, Brazzaville, Republic of Congo; 5 Institute for Tropical Medicine, University of Tübingen, Tübingen, Germany

**Keywords:** *Plasmodium falciparum*, Malaria, Multiplicity of infection, Genetic diversity, Clinical episodes, *msp2* gene

## Abstract

**Background:**

In this first study conducted after the introduction of artemisinin-combination therapy (ACT), the major objective was to evaluate *Plasmodium falciparum* genetic diversity and multiplicity of infection in isolates from Congolese children between one and nine years of age enrolled and followed up for one year. The secondary objective was to characterize the *msp2* profiles of *P. falciparum* isolates collected from successive malaria episodes in ten children who had four or more clinical episodes during the follow up.

**Methods:**

Three-hundred and thirteen children residing in southern part of Brazzaville participated in this study. Blood samples were obtained from all children at enrollment and checked for *P. falciparum* infection. Based on the one year follow-up data, two clinical groups were considered according to the number of malaria episodes presented over the follow up period: “protected”(children who did not experience any episode) and “unprotected” (those who experienced more that two episodes). Therefore, the *msp2* genetic diversity of *P. falciparum* isolates collected at enrollment in the two groups was characterized by allele-specific nested PCR and compared. The *msp2* profiles of *P. falciparum* isolates collected from successive malaria episodes was also characterized by allele-specific nested PCR.

**Results:**

Forty-three percent of FC27 and fifty-seven percent of 3D7 in protected vs fifty-six percent of FC27 and forty-four percent of 3D7 in isolates from unprotected children were detected. Seven and two alleles belonging to the FC27, and six and three alleles belonging to 3D7 families were distinguished in isolates from protected and unprotected children respectively. The mean multiplicity of infection (MOI) values at inclusion for the *msp2* locus was 1.29 and 1.43 for protected and unprotected children respectively. 43 isolates were obtained from the ten children who had four or more clinical episodes during the follow up. A total of 63 alleles or fragments corresponding to 57% (36/63) FC27 and 43% (27/63) 3D7 were detected. The variant 400bp of FC27 was the most prevalent. 46% (20/43), 42% (18/43), 2% (1/43) and 2% (1/43) of isolates were found to have 1, 2, 3 and 4 parasite genotypes respectively and the mean MOI was 1.78.

**Conclusion:**

This study shows that the introduction of ACT in the Republic of Congo has reduced the MOI but not the genetic diversity of *P. falciparum* isolates from children living in Southern districts of Brazzaville.

## Background

The last decade, tremendous concerted efforts have been made to control malaria [1], and the success observed in some countries (Tanzania, Ethiopia) has raised hope in the global community. This decline in the disease burden with 655,000 deaths in 2011 could be attributed to control interventions like insecticide-treated nets, indoor insecticide spraying, deployment of artemisinin-combination therapy (ACT) and intermittent preventive treatment for pregnant women [2,3].

The challenges facing all those fighting malaria are the elaboration of efficient tools that could be used in any endemic country and reached all the affected population. In that perspective, malaria vaccine would be the best weapon if efficient and affordable by poor people [4,5].

In areas where malaria is endemic, immunity to *Plasmodium falciparum* malaria develops slowly and is hardly ever complete. This phenomenon is explained by the fact that many parasite strains, differing in the sequences of key protective antigens circulate within any given malaria endemic area [6]. The genetic and antigenic diversity of *P. falciparum* strains has been reported as a major obstacle for the development of an effective malaria vaccine. Indeed, it has been shown geographical diversity of *P. falciparum* strains in asymptomatic and symptomatic isolates [7] and it is quite difficult to identify the best immune targets that will lead to the malaria vaccine, eventually. There is a need to understand why some children do not develop malaria episodes while others have repeatedly malaria attacks under the same malaria and socio-economic exposure. Human genetic background like sickle cell trait carriage, blood group and other mutations have been reported to influence susceptibility to clinical malaria [8,9] of some exposed individuals but the mechanisms remain poorly understood.

The merozoite surface protein−2 (MSP-2) of *P. falciparum* is considered as a good candidate for inclusion into a malaria vaccine. Several studies have reported the relationship between protection and humoral immune responses to MSP-2 antigens [10,11]. Moreover, merozoite surface protein-2 gene (*msp2*) is also commonly used as a single marker for the molecular characterization of field malaria parasites [12], because it appears to be more or at least as equal reliable as the merozoite surface protein-1 (*msp1*) marker [6,13]. These genes are represented as a single copy on *P. falciparum* genome [14] and high degree of polymorphism has been reported in the block 2 for *msp1* gene [15] and in the central variable region for *msp2* gene [14]. Typing of these different polymorphic *P. falciparum* genome regions have permitted to determine malaria infection indicators e.g. diversity of *P. falciparum* strains and multiplicity of infection (MOI), which may contribute to the description of malaria situation in a given location.

Against this background, many sub-Saharan African countries cannot, because of limited resources, report updated data on the malaria situation and specifically on the genetic diversity of malaria parasites circulating in their areas. These data would assist in identifying the most appropriate strategies for control and also to evaluate the impact of control interventions.

In the Republic of Congo, since 2009, the Central Africa Network on Tuberculosis, HIV/AIDS and Malaria (CANTAM) has initiated baseline epidemiological studies for collecting *in vivo* and *in vitro* data on sensitivity to anti-malarials and characterization of malaria parasites infecting children living in Brazzaville. The major objective of the present study carried out in the southern part of Brazzaville was to evaluate *P. falciparum* genetic diversity and multiplicity of infection in isolates from children who did not have any malaria episode (considered as “protected” against clinical malaria) and children who presented three or more uncomplicated malaria episodes (considered as “non-protected”) during one year follow-up. The secondary objective was to characterize the *msp2* profiles of *P. falciparum* isolates collected from successive malaria episodes in ten children who had four or more clinical episodes during the follow up.

## Methods

### Study site

The study was conducted in Madibou, a semi-urban district located in a Southern part of Brazzaville [16], where malaria transmission is high and perennial with an entomological inoculation rate (EIR) of 22.5 infective bites/person/year estimated several years ago [17,18].

### Ethical clearance

This study was approved by the Institutional Ethics Committee for Research on Health Sciences. Written informed consent was given by parents or guardians of children who participated in the study.

### Study population

Children between one and nine years of age and permanent residents of the study area were enrolled in a cohort study for malaria surveillance [19]. Inclusion criteria were absence of clinical malaria in the last two weeks and at least one week after enrolment, and with an axillary temperature of <37.5°C and parasite density <5,000 parasites/μl of blood. Recruitment was done from April to June 2010. At inclusion, after clinical examination by the physician, thick and thin blood smears were made from each child and whole blood were also collected for subsequent analyses including DNA extraction and haemoglobin genotyping. Parasite density was determined and genomic human and parasite DNA were analysed as described by Koukouikila-Koussounda *et al.*[19]. Then, children were passively followed up during one year. Children with fever or any disease symptoms were asked to go to the health care, where appropriate standard care were provided. In case of malaria, a blood sample was collected, parasite density determined and treatment with artesunate-amodiaquine or artemether-lumefantrine provided by the clinician. During the follow-up, 141 children did not present any clinical malaria episode and, although it is known that there is no sterile protection against malaria, these children were considered as “protected”. Other children presented several malaria attacks and were considered as “unprotected”.

#### *Plasmodium falciparum msp2* genotyping

Genotyping of *P. falciparum* parasites was performed with nested PCR assays based on the amplification of *msp2* as described in details elsewhere [20]. The variable block 2 was amplified to distinguish allelic families 3D7 and FC27. The amplified products from the nested reaction were separated using 2% agarose gel (PeqLab, Erlangen, Germany), and visualized under ultraviolet (UV) trans-illumination. A 100 base pairs (bp) DNA ladder marker (Invitrogen, Karlsruhe, Germany) was used to determine the size of bands. The size polymorphism in each allelic family was estimated assuming that one band represented one amplified PCR fragment derived from a single copy of *msp2* gene. Determination of haemoglobin type (AA, AS and SS) in this cohort of children was performed as described by Koukouikila-Koussounda *et al.*[19].

#### *Plasmodium falciparum msp2* allele types distribution and multiplicity of infection

The prevalence of the different *msp2* alleles was determined as the presence of PCR products in the total number of *msp2* amplified bands. The MOI was defined as the mean number of *P. falciparum* genotypes per infected individual. It was calculated as the quotient of the total number of *P. falciparum msp2* genotypes and the number of PCR positive samples.

### Statistical analysis

Statistical analysis was performed using R software, version 2.12. Frequency of *msp2* allelic families in both groups (“protected” vs “unprotected”) was compared using a Chi 2 test. Comparisons of the geometric mean parasite density (GMPD) between the two groups as well as that of the MOI were made using the Kruskal-wallis tests. Differences were considered statistically significant at *P* value < 0.05.

## Results

### Description of the recruited children

Of the 313 samples collected at inclusion, 28 were detected with *P. falciparum* parasites representing 9% and 24 sub-microscopic infections were detected by PCR. Therefore, a prevalence of 16% (52/313) of *P. falciparum* infections was found. *Plasmodium falciparum* was the only species detected. Children enrolled in this study were aged from one to nine years old and were grouped into three groups (0, 1 to 2, ≥3 according to the number of malaria attacks presented during the one year follow-up). It was found that 10 children had more than four malaria attacks. As defined in the Methods section, children who did not present any malaria attacks were considered as “protected” against clinical malaria and those with more than two clinical episodes as unprotected (Table 1). The GMPD was found to be significantly different between these two groups, being lower in the group of protected children and higher in the group of unprotected children (*P*=0.01).

**Table 1:** Description of patients

**Cases (n)**	**Age median ± SD**	**Number of positive microscopic cases**	**GMPD (p/μl)**	**Number with sub-microscopic infection (PCR+)**	**Overall asymptomatic infections prevalence (%)**	**Number of children carrying Hb AA**	**Number of children carrying HbAS**
**Protected (141)**	3 ± 2.57	9	684*	9	12.76	111	30
**1 to 2 malaria attacks (141)**	5 ± 2.55	14	1,310	13	19.15	119	21
**Unprotected (31)**	5 ± 2.33	5	3,890*	2	22.58	23	7
***P-value***	NS	0.007	NS	NS	NS		

*p-value = 0.01.

### Genetic diversity and allelic frequency

**Figure 1:**
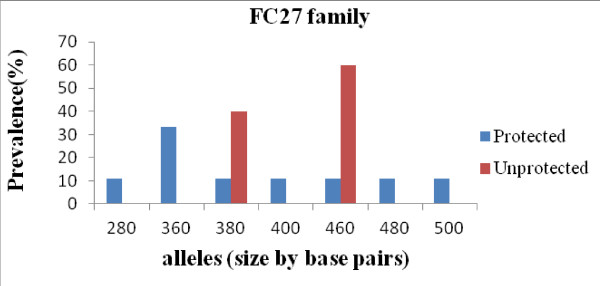
**Distribution of FC27 alleles of *P. falciparum msp2 *gene in isolates from Congolese children at enrollment.**

**Figure 2:**
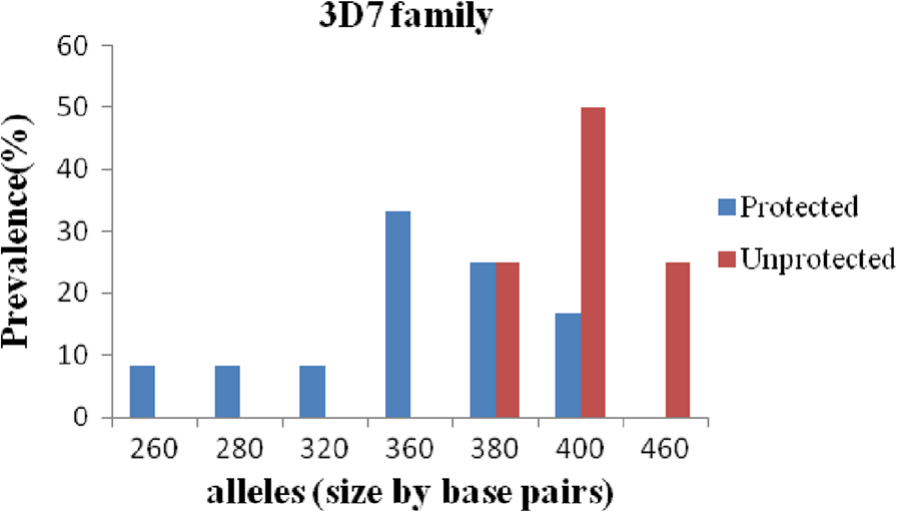
**Distribution of 3D7 alleles of *P. falciparum msp2* gene in isolates from Congolese children at enrollment.**

The band sizes of *msp2* FC27 alleles ranged from 260 to 540 bp and that of 3D7 alleles ranged from 180 to 480 bp. The prevalence of FC27 and 3D7 family represented 45% and 55% respectively in overall isolates from asymptomatic children. Considering the two groups, protected or unprotected, we found 43% FC27 and 57% 3D7 in protected vs 56% FC27 and 44% 3D7 in isolates from unprotected children. 7 and 2 alleles belonging to the FC27 family, and 6 and 3 alleles belonging to the 3D7 family were distinguished in isolates from protected and unprotected children respectively as shown in Figures 1 and 2.

### Multiplicity of *P. falciparum* infection

The mean MOI values at inclusion for the *msp2* locus were 1.29 and 1.43 for protected and unprotected children respectively (p<0.047).

### Characterization of *P. falciparum* isolates from successive malaria episodes

The 10 children who presented four or more clinical malaria episodes during the follow up were selected for the characterization of all isolates collected during these attacks. The geometric mean parasite density from all successive malaria episodes was 22,964 parasites *per* microliter of blood (p/μl), ranging from 160 to 473,882 p/μl. A total of 43 samples were analysed. In order to distinguish between recrudescence and new infection when two malaria episodes occurred at a too short period (less than 45 days), two additional *P. falciparum* molecular markers were used, *msp1* and glutamate-rich protein (*glurp*). No recrudescent pattern was found. Using *msp2* marker, a total of 63 alleles or fragments corresponding to 43% 3D7 and 57% FC27 were detected from the 43 different episodes. A number of 27 (11 different allele types) and 36 (10 different allele types) fragments were identified to belong to 3D7 and FC27 allelic families respectively. However, the variant 400bp of FC27 was the most prevalent in clinical isolates (Table 2).

**Table 2:** **Profiles of *P. falciparum msp2 *genotypes during successive episodes**

	**Episode1**	**Episode2**	**Episode3**	**Episode4**	**Episode5**	**Episode6**
Child1	A_600_	A_480_	A_500_+B_300_	B_280_		
Child2	A_600_+B_400_	A_380_+A_480_	--	A_500_		
Child3	A_500_	A_500_	A_480_+A_520_	B_380_	B_380_	B_500_
Child4	A_300_	A_350_	A_400_	A_300_+B_280_		
Child5	A_380_	A_400_+B_300_	B_180_	--		
Child6	A_280_+A_300_+B_380_+B_400_	A_280_+B_450_	A_350_+A_400_	A_300_		
Child7	A_400_+B_450_	A_300_+B_420_	A_300_+B_450_	B_300_		
Child8	A_350_+B_250_	A_350_+B_300_	A_320_+B_280_	B_220_+B_450_		
Child9	A_350_	A_400_+B_350_	A_400_+B_300_	A_450_		
Child10	--	B_380_	A_300_+B_300_	A_300_+A_400_+B_300_	B_320_	

Note: A and B represent alleles for FC27 and 3D7 families respectively.

With regard to the number of isolates containing one, two, three and four different genotypes, 20/43 (46%), 18/43 (42%), 1/43 (2%) and 1/43 (2%) were found to have one, two, three and four parasite genotypes, respectively. Three samples were not identified to any of the 3D7 or FC27 allelic family despite being repeatedly amplified. This might be due to a mutation in the annealing region of the primers. The mean MOI for these ten children was 1.0 at inclusion (asymptomatic phase) and 1.78 for the all clinical episodes.

## Discussion

In malaria endemic areas, people are exposed to diverse *P. falciparum* strains and this contributes to the development of natural immunity including clinical and parasite-immunity. Determining *P. falciparum* genetic diversity and MOI from field samples may be useful and helpful indicators to describe malaria infection in a place and to relate to the level of immunity at the time of infection [21].

This study carried out in 2009 in Southern part of Brazzaville after the introduction of ACTs showed that *P. falciparum* genetic diversity in isolates from Congolese children did not change and remains at about 20 *msp2* alleles. To the best of our knowledge, this study is the first to describe *P. falciparum* infections in clinical cases in Congolese children after the introduction of ACTs in the Republic of Congo. Based on the one year follow up of children, two clinical groups were considered according to the number of malaria episodes presented over the study period: “protected” and “unprotected” referring to the absence of malaria episodes or more than two malaria episodes respectively. Interestingly, *msp2* genetic diversity in the “protected” group was higher compared to the unprotected children. A possible explanation could be the better control of *Plasmodium falciparum* strains by protected children under a threshold leading to fever [22].

The genetic diversity of the *msp2* is limited in both clinical groups at asymptomatic phase. However, it is observed that the 3D7 allelic family, which is the most prevalent in the general population [19] is also the most prevalent in protected children, whereas the FC27 allelic family predominated in the unprotected group, pointing out that FC27 allelic types are the less successful controlled strains in unprotected children and probably acquisition of natural semi-immunity in this population has to include the control of these specific strains. In many countries (Tanzania, Burkina Faso, Malawi and Uganda), Mwingira *et al.*[23] showed that the 3D7 family was the most predominant in clinical isolates. Contrary, in Gabon [24] and Cameroon [25], the FC27 family was found to be the most predominant parasite allelic types. This difference can be explained by the fact that genetic diversity of *P. falciparum* differs according to geographic areas, and the level of transmission [26].

With regard to the parasite density, during the asymptomatic phase, a significantly higher parasite density was observed in unprotected children. These findings are in agreement with another study suggesting the association of asymptomatic parasitaemia of higher parasite density with a higher risk of symptomatic malaria in children [25]. Considering the molecular epidemiological studies carried out in this population before the introduction of ACT [27,28], the level of parasitaemia in clinical cases is reduced in the present study. This suggests that intermittent treatment of asymptomatic infections with a high parasitaemia would be an efficient tool of preventing clinical episodes of malaria in childhood. To implement such an intervention, the definition of a parasitaemia threshold for Congolese patients [22], which has not been defined, would be imperative.

The MOI is considered to be a key indicator of malaria infection in humans and may reflect to some degree malaria transmission and immunity [6]. The mean MOI was slightly higher in unprotected children and this result correlates with the higher parasite density in unprotected children with asymptomatic infection. This finding could be interpreted as a reduce acquired immunity in unprotected children confirming a higher risk of occurrence of clinical malaria [6,21,29]. However, the mean MOI in clinical isolates is lower than expected from previous studies conducted in the same area and the same age group. It is important to note the low MOI of about 1.3 and the high number of children harbouring one malaria genotype in the study group with regard to the level of malaria transmission [19] This is different to what was reported from other endemic areas in Central Africa [23,30]. This could be explained by the massive distribution of impregnated mosquito nets by the government and supporting agencies and the deployment of artemisinin-combination therapy in the country. As a result, there has been a decrease of the burden of malaria parasites, reflected in lower parasite densities, but this did not influence the diversity of parasites in circulation.

In a second step of this study, *P. falciparum* infections during successive clinical attacks in unprotected children have been characterized by analysing each isolate using three molecular markers: *msp1*, *msp2* and *glurp* when necessary [23]. The discrimination between recrudescence and new infections has been carefully analysed. It appears that the successive clinical episodes experienced by each child were caused by genetically distinct parasite populations. This gives a confirmation that each episode was a true one instead of being a recrudescence and only one child for one episode was identified as recrudescence. Therefore, we can claim that malaria episodes in these children were caused by new inoculated parasites. These findings are in line with previous reports from Soudan, Senegal, and Gabon [31-33]. It is worth noting that the FC27 fragment of 400bp, which was the most prevalent in clinical episodes of malaria, was also observed with a high frequency in other studies among symptomatic malaria children [34,35]. This may indicates an association between this FC27 allelic type and clinical episodes, hence predicts a possible candidate antigene that can be considered in designing malaria vaccine.

As a conclusion, this study shows that the introduction of ACT in the Republic of Congo has reduced the multiplicity of infection but not the genetic diversity of *P. falciparum* isolates from children living in Southern districts of Brazzaville. It also points out that children exposed to the same malaria transmission and socio-economic conditions might have different susceptibility to malaria infections. The two groups described here are important for designing additional studies to investigate human and parasite genetic factors that may be involved in the susceptibility/resistance to malaria in this area.

## Abbreviations

MSP-2: Merozoite surface protein-2; *msp2*: Merozoite surface protein-2 gene; *msp1*: Merozoite surface protein-1 gene; PCR: Polymerase chain reaction; EIR: Entomological inoculation rate; UV: Ultra-violet; bp: Base pair; MOI: Multiplicity of infection; GMPD: Geometric mean parasite density; p/μl: Parasite *per* microliter; *glurp*: Glutamate rich protein gene; ACT: Artemisinin combination therapy.

## Competing interests

The authors declare that they have no competing interest.

## Authors’ contributions

RIO participated in genomic DNA extraction, molecular genetic study and writing of the manuscript. FKK contributed in data analysis and writing of the manuscript. CJV participated in data analysis. VM participated in molecular genetic study. MN and NPC designed and supervised the field work. AS contributed in writing of the manuscript. JRI participated in correction of the manuscript. FN supervised the different steps of the work and participated in writing of the manuscript. All authors contributed to the final manuscript.
